# Esophageal pulmonary fistula – a rare complication of radiation therapy: a case report

**DOI:** 10.1186/s13256-018-1658-3

**Published:** 2018-05-02

**Authors:** Laetitia Buemi, Salvatore Stefanelli, Philippe Bichard, Mickaël Luscher, Minerva Becker

**Affiliations:** 1 Division of Radiology, Department of Imaging and Medical Informatics, Geneva University Hospitals, Rue Gabrielle-Perret-Gentil 4, 1211 Geneva 14, Switzerland; 2 Clinic of Gastroenterology and Hepatology, Department of Medical Specialties, Geneva University Hospitals, Geneva, Switzerland

**Keywords:** Esophageal respiratory fistula, Esophageal pulmonary fistula, Squamous cell lung carcinoma, Postradiotherapy, Videofluoroscopy

## Abstract

**Background:**

Esophageal respiratory fistulae are abnormal communications between the esophagus and the respiratory system. They are either congenital or acquired. Most acquired esophageal respiratory fistulae are of the esophageal tracheal and esophageal bronchial type and are caused by infections or malignant neoplasms, whereas esophageal pulmonary fistulae are rare.

**Case presentation:**

We report a case of a 72-year-old Caucasian man with squamous cell carcinoma of the lung presenting with abrupt-onset dyspnea during localized mediastinal radiotherapy. His laboratory test results suggested major respiratory infection. A chest x-ray revealed left apical lung radiopacity along with excavated lesions, consistent with secondary tumor infection. No clinical improvement was observed despite antibiotic treatment. A contrast-enhanced computed tomographic scan of the chest confirmed persistent lung infection with unfavorable progression and air in the mediastinum; the latter suggested a fistula from the upper third of the esophagus to the upper left pulmonary lobe. Videofluoroscopy confirmed the diagnosis of an acquired esophageal pulmonary fistula. The patient underwent endoscopy, and an esophageal self-expandable metallic stent was deployed.

**Conclusions:**

Esophageal pulmonary fistulae must be suspected whenever patients undergoing local mediastinal radiotherapy present with acute pulmonary complications, particularly pneumonia resistant to antibiotic treatment. Esophageal pulmonary fistulae are diagnosed by means of radiological imaging. Because esophageal respiratory fistulae are acute life-threatening conditions, prompt treatment with an endoscopically placed covered stent proves vital.

## Background

Acquired esophageal respiratory fistulae (ERFs) of the esophageal tracheal or esophageal bronchial type are well-recognized complications of malignant neoplasms, infections, traumatic lesions, or surgery of the esophagus or tracheobronchial tree [1]. On the contrary, esophagopulmonary fistulae are very uncommon life-threatening conditions. In this report, we present a case of an esophagopulmonary fistula occurring during mediastinal radiotherapy. The diagnosis was suspected on the basis of chest computed tomography (CT) and was subsequently confirmed by means of an X-ray videofluoroscopic swallowing study (videofluoroscopy).

To the best of our knowledge, esophageal pulmonary fistulae occurring during radiotherapy for lung cancer have not been previously described. The aim of this case report is to make readers aware of this rare, life-threatening complication of treatment, which occurred during mediastinal radiotherapy and which mimicked clinically and radiologically classic pulmonary infection. We equally aim to highlight the role of videofluoroscopy as a noninvasive and cheap diagnostic tool allowing the correct diagnosis; this diagnosis can be overlooked, misinterpreted, or only suspected when using static imaging modalities such as CT. Therefore, whenever an ERF is suspected, videofluoroscopy should be performed.

## Case presentation

A 72-year-old Caucasian retired man with a 100 pack-year smoking history who was a married father of three children underwent medical investigation because of progressively increasing dysphagia, acute dysphonia, and a 3-kg weight loss. His medical history revealed a left testicular seminoma treated by orchiectomy and radiotherapy 12 years previously with complete remission, as well as long-standing epilepsy, hypertension, and tendinopathy of the rotator cuff. His clinical examination revealed hoarseness and a palpable mass in the left supraclavicular fossa. The results of cardiopulmonary auscultation and a neurological examination were within the normal limits. The patient’s vital signs were normal. The results of his laboratory tests, including complete blood count and liver and renal function, were within the normal limits, except for his red blood cell count, which revealed anemia with 129 g/L hemoglobin (reference range 140–180 g/L).

**Fig. 1:**
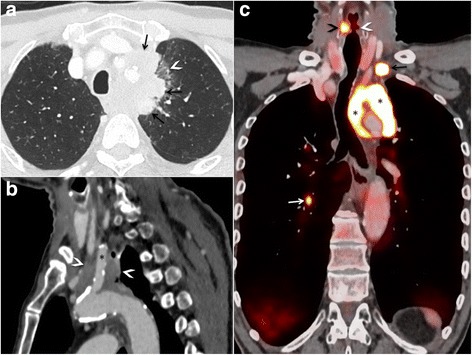
(**a**) Axial contrast-enhanced computed tomographic (CT) scan (lung window) depicts an upper left mediastinal mass (*arrows*) infiltrating the left upper lobe, which displays a “ground-glass opacity” (*arrowhead*). (**b**) Sagittal contrast-enhanced 2D reconstruction (soft tissue window) shows local tumoral extension (*arrowheads*) surrounding the aortic arch and origin of the left common carotid artery (*asterisk*). Both arteries are patent. (**c**) Coronal contrast-enhanced positron emission tomographic-CT scan reveals left upper lobe and mediastinal fludeoxyglucose (^18^F-FDG) uptake (*asterisks*). Hypermetabolic left supraclavicular lymph node (*black arrow*) and hypermetabolic pulmonary nodule in the right lower lobe (*white arrow*). Indirect signs of left recurrent laryngeal nerve palsy are present in terms of enlarged left laryngeal ventricle (*white arrowhead*) along with compensatory increased ^18^F-FDG uptake of the right vocal cord (*black arrowhead*)

Contrast-enhanced positron emission tomography (PET) with fludeoxyglucose was performed (Fig. 1), which demonstrated a hypermetabolic mass in the left upper lung lobe associated with mediastinal infiltration. PET-CT likewise revealed a 7-mm hypermetabolic nodule in the contralateral lower lobe, along with mediastinal and left supraclavicular lymphadenopathy (short axis < 15 mm). Indirect signs of left recurrent laryngeal nerve paralysis secondary to the mediastinal mass were identified as causing hoarseness. A bronchoscopic biopsy obtained 1 day after PET-CT confirmed squamous cell carcinoma of the lung (Union for International Cancer Control TNM classification cT4N3M1).

A partial oncologic response was seen after six chemotherapy cycles with carboplatin-gemcitabine over a 4-month period, with an overall decreasing size of the pulmonary mediastinal tumor from 58 × 44 mm to 31 × 26 mm on axial CT images, consistent with a > 30% reduction of the longest diameter of the target lesion, according to RECIST 1.1 criteria (Response Evaluation Criteria in Solid Tumors) [2].

Local radiotherapy was initiated 6 weeks thereafter. After three sessions of 2.15-Gy radiation doses, each on the left paramediastinal region and apical segment of the left upper lobe, the patient was hospitalized because of major dyspnea. Upon admission, clinical examination revealed bilateral rhonchi, and neurological examination yielded 12 points on the Glasgow Coma Scale with a decorticate response to pain stimuli. The rest of the results of the clinical examination were within normal limits. Oxygen saturation was decreased to 70%. The patient’s heart rate was 130 beats/minute, body temperature was 39.7 °C, and blood pressure was 113/75 mmHg. His laboratory test results were suggestive of major infection with leukocytosis at 19,000/L (reference range 4,000–11,000/L), and his C-reactive protein concentration was 415 mg/L (reference range 0–10 mg/L). His complete blood count revealed moderate anemia with 98 g/L hemoglobin (reference range 140–180 g/L). His liver and renal function test results were within the normal limits. Arterial blood gas analysis confirmed respiratory acidosis with a pH of 7.18 (reference range 7.35–7.45), arterial partial pressure of carbon dioxide of 8.7 kPa (reference range 4.67–6.4 kPa), and arterial partial pressure of oxygen of 8 kPa (reference range 11.07–14.4 kPa). The results of the patient’s urine analysis were normal. At admission, the patient was receiving the following medications: irbesartan for arterial hypertension, clonazepam, and valproic acid for epilepsy, and ibuprofen and tizanidine for tendinopathy of the rotator cuff.

**Fig. 2:**
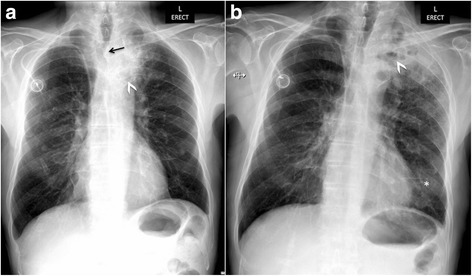
(**a**) Initial chest x-ray reveals an apical left lung partially excavated opacity (*arrowhead*) associated with a silhouette sign of the upper left mediastinum, consistent with secondary tumor infection. Note that owing to mass effect, there is right-sided tracheal deviation (*arrow*). (**b**) A control chest x-ray obtained 10 days later shows an unfavorable evolution as seen by an increased infectious tumor focus, progression of the excavated components (*arrowhead*), and occurrence of lingular opacity (*asterisk*)

**Fig. 3:**
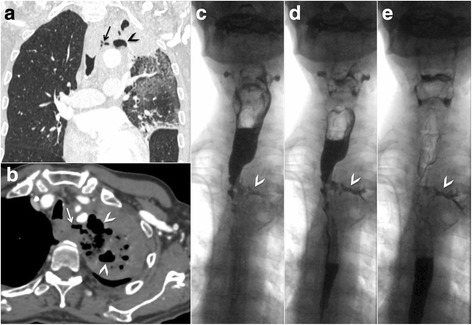
**a**, **b** Coronal and axial thoracic contrast-enhanced computed tomographic scans (lung and soft tissue window, respectively) show a condensation of the apical segment of the left upper lobe with parenchymal excavations (*arrowheads*) and extended pulmonary infiltrations involving the whole lobe. A fistula between the esophagus and left upper lung is suspected due to the presence of air in the mediastinum (*arrows*). **c**–**e** Videofluoroscopic studies showing contrast leakage through a fistulous tract (*arrowheads*) originating from the upper esophagus and progressively joining the left apical lung without contrast bronchogram, thus confirming the esophagopulmonary type of fistula

A chest x-ray (Fig. 2a) revealed a left superior mediastinal pulmonary mass and parenchymal radiopacity in the left apical lung, along with excavated lesions and a “silhouette sign” of the left mediastinum, consistent with secondary infection. The patient’s blood test results were positive for gram-positive cocci. The patient was treated with intravenous antibiotics (piperacillin/tazobactam 4.5 g every 8 hours and vancomycin 1 g every 12 hours) for severe pulmonary sepsis. Despite antibiotic treatment, his fever and dyspnea persisted over the 10-day treatment period. A repeat chest X-ray revealed multiple excavated lesions of increasing size (Fig. 2b). A contrast-enhanced CT scan was then obtained (Fig. 3), which confirmed persistent lung infection with unfavorable extensive evolution. Moreover, a fistula from the upper third of the esophagus to the left upper pulmonary lobe was suspected on the basis of the presence of air in the mediastinum. To confirm the suspected esophageal pulmonary fistula, videofluoroscopy with a water-soluble contrast agent was performed (Fig. 3). This clearly depicted leakage of contrast material from the cervical esophagus into the left lung via a fistulous tract.

**Fig. 4:**
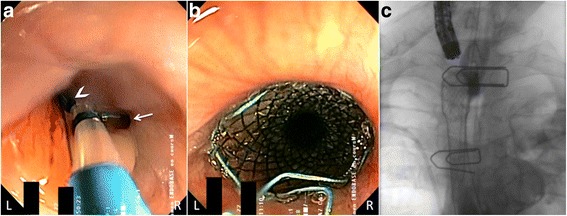
(**a**) Endoscopic view of the upper esophagus visualizes the origin of the esophagopulmonary fistula (*arrowhead*) on the left site of the esophagus lumen (*arrow*). (**b**) Endoscopic view after treatment illustrates how the fistula was managed with a fully covered self-expandable metallic stent. (**c**) Radioscopic control after stent deployment proves the fistulous tract to be excluded, with no esophagopulmonary contrast leakage

Two days later, the patient underwent endoscopy, thereby allowing fistula treatment with a covered self-expandable metallic stent, placed just below the upper esophageal sphincter (Fig. 4). The patient recovered from his pulmonary infection within 2 weeks after fistula treatment. Four weeks later, a CT scan was obtained because of hemoptysis. The CT images showed a major decrease of the left upper lung lobe infection but progression of the mediastinal tumor with vascular encasement, in particular invasion of the left common carotid artery, left subclavian artery, and left brachiocephalic vein. One week after a percutaneous embolization attempt, the patient died of massive hemoptysis. No autopsy was performed.

## Discussion

Our patient was a 72-year-old man with squamous cell carcinoma of the lung who underwent chemotherapy followed by localized mediastinal radiotherapy. During radiotherapy, the patient developed pulmonary sepsis. Despite antibiotic treatment, his clinical situation did not improve. CT revealed pulmonary infection, and an ERF was suspected. To confirm and further clarify the diagnosis, videofluoroscopy was performed, which clearly showed an esophageal pulmonary fistula. The patient underwent endoscopic treatment with a covered self-expandable metallic stent.

This report demonstrates a rare and devastating complication occurring during radiotherapy in a patient with lung cancer. Owing to its very rare occurrence in the setting of a routine treatment protocol, the diagnosis of esophageal pulmonary fistula may be delayed, thereby leading to life-threatening complications. Because the clinical symptoms are indistinguishable from pneumonia complicating standard treatment, a more aggressive diagnostic approach, including CT and, whenever necessary, videofluoroscopy is warranted.

As their name suggests, ERFs are defined as abnormal communications between the esophagus and respiratory system, either of congenital or acquired etiology [1]. Congenital fistulae are mostly diagnosed in the neonatal period, whereas acquired fistulae are more commonly detected in adults [3]. Of all ERFs, the esophagotracheal subtype is the most common (52–57%), followed by the esophageal bronchial (37–40%) and esophageal pulmonary (3–11%) subtypes [4]. Nontumoral causes include infections, such as granulomatous mediastinal diseases, or traumatic lesions, such as iatrogenic complications after prolonged intubation, upper respiratory and digestive endoscopic procedures or surgical interventions, as well as Crohn’s disease [1, 3, 5, 6].

Esophageal, lung, or mediastinal cancers are the main malignancies responsible for ERFs [1]. Approximately 15% of tracheal cancers, 5% of esophageal cancers, and 0.2% of lung malignancies have been reported as causing esophageal pulmonary fistulae [5]. Radiotherapy has also been described as a late cause of ERF [1, 5, 7]; however, to the best of our knowledge, esophageal pulmonary fistula occurring during radiation therapy for lung cancer has not been described so far. Radiotherapy complications in lung cancer typically include radiation pneumonitis and pulmonary fibrosis, as well as cardiac toxicity [8]. Likewise, esophageal radiotherapy complications include esophagitis as an early side effect, strictures as a late side effect, and esophageal cancers as late radiation-induced malignancies [9].

Three mechanisms explain dysphagia associated with lung cancer: (1) extrinsic esophageal tumor compression, (2) compression of the upper esophagus by neck lymphadenopathy, and (3) esophageal stenosis secondary to local radiotherapy [10]. In patients with prior lung or esophageal cancer, the association of typical symptoms such as dysphagia or pulmonary complications such as recurrent or treatment-resistant pneumonia should raise the issue of whether there is an underlying fistula [1, 5].

The accurate investigation of fistulae should comprise CT scanning and videofluoroscopy, the latter being particularly useful to detect cases that have been misdiagnosed on the basis of CT scans [1]. As an entirely noninvasive and cheap diagnostic procedure, videofluoroscopy can easily be carried out even in patients with limited cooperation. To prevent complications such as pulmonary acute edema or mediastinitis, thin-slice thoracic CT scans and videofluoroscopy must be performed with a nonirritating, water-soluble, and low-osmolar oral contrast agent in order to detect fistulae in a safe manner. Furthermore, endoscopy allows for these conditions to be satisfactorily managed.

Fistulae may prove life-threatening [11]. In the absence of appropriate treatment, they can lead to pneumonia, lung abscess, and eventually sepsis or acute respiratory distress syndrome [1]. Early treatment is mandatory for malignant fistulae and relies on palliative treatment such as stenting, which provides immediate symptom relief [1, 4, 11–13]. Fully covered self-expandable metallic stents have recently emerged as a superior treatment in managing fistulae owing to their high efficiency [10, 11, 13]. These devices are recommended by the European Society of Gastrointestinal Endoscopy and are indicated in the event of dysphagia, esophageal compression, or lung cancer infiltrating the esophagus [12, 13]. Their success rate varies from 70% to 100%, depending on the report [11, 13]. Complications such as stent migration, bleeding, granulation formation, and foreign body sensation are well known [10, 13, 14]. Secondary fistulae have been reported to be a late complication of stenting, occurring in about 10% of cases [12–14].

Our patient was undergoing localized mediastinal radiotherapy treatment when he developed severe pneumonia. Although the first CT scan did not show any fistulous tract, our suspicion was raised by the CT scan obtained after the third localized radiotherapy session. This reinforced our hypothesis of the fistula being a possible consequence of treatment.

## Conclusions

An acquired esophageal pulmonary fistula is a rare but life-threatening complication of standard radiotherapy. It should be suspected whenever patients develop clinical symptoms of severe lung infection while undergoing mediastinal radiotherapy, and in particular in the event of antibiotic resistance. The diagnosis relies mainly on radiologic examinations, such as CT and videofluoroscopy. Because CT may fail to depict the fistula, videofluoroscopy should be carried out. Videofluoroscopy is a cheap and noninvasive diagnostic modality allowing the definitive and correct diagnosis. Early detection and adequate treatment are essential for patient symptom relief and survival.

## Abbreviations

^18^F-FDG: ^18^F-fludeoxyglucose
CT: Computed tomography
ERF: Esophageal respiratory fistula
PET: Positron emission tomography
RECIST: Response Evaluation Criteria in Solid Tumors
